# Precision Microbiome Interventions in Oncology: From Predictive Biomarkers to Therapeutic Modulation of Immunotherapy Outcomes

**DOI:** 10.3390/microorganisms14102147

**Published:** 2026-09-23

**Authors:** Manuele Biazzo, Marco Soriani, Maria Pia Micallef, Eman Zaky, Christine Podrini

**Affiliations:** 1 Laboratories at Life Science Park (LS2.1.10, LS2.1.12-LS2.1.15), The BioArte Ltd., Triq San Giljan, SGN 3000 San Gwann, Malta; m.biazzo@thebioarte.com (M.B.); m.micallef@thebioarte.com (M.P.M.); zakyeman@gmail.com (E.Z.); 2 Independent Researcher, Viale G. Matteotti 93, 58022 Follonica, Italy; soriama6@gmail.com

**Keywords:** gut microbiome, cancer immunotherapy, immune checkpoint inhibitors, faecal microbiota transplantation, live biotherapeutic products, precision oncology, microbial metabolites, predictive biomarkers, microbiome modulation

## Abstract

Immune checkpoint inhibitors (ICIs) have transformed the treatment of multiple malignancies, yet durable clinical responses remain limited to a subset of patients. Accumulating evidence identifies the gut microbiome as a regulator of antitumour immunity that can influence treatment efficacy and toxicity through microbial metabolism, epithelial barrier integrity, inflammatory signalling, and host immune responses. This review critically evaluates the microbiome as both a predictive biomarker and therapeutic target in precision oncology. It moves beyond descriptive taxonomic associations by assessing emerging functional and mechanistic models that integrate microbial metabolites, ecological interactions, strain-level variation, and host–microbiome communication. Clinical and translational evidence from melanoma, non-small cell lung cancer, colorectal cancer, hepatocellular carcinoma, renal cell carcinoma, and urothelial carcinoma reveals marked differences in the reproducibility and clinical maturity of reported microbial signatures. Emerging microbiome-targeted strategies, including dietary modulation, faecal microbiota transplantation (FMT), live biotherapeutic products (LBPs), and defined microbial consortia, may enhance ICI responsiveness, with responder-derived FMT providing important early clinical proof of concept. However, inter-individual variability, donor–recipient compatibility, microbial engraftment, the lack of standardised analytical approaches, and unresolved safety and regulatory requirements continue to limit clinical translation. Pending prospective validation, reasonable clinical practice is to avoid unnecessary microbiome-disrupting antibiotics, systematically document diet and concomitant medications, evaluate FMT and LBPs within regulated clinical trials, and interpret microbiome profiles alongside established tumour and host biomarkers rather than as stand-alone tests. Current evidence therefore supports a cautious shift towards functional microbiome-guided precision oncology, providing a foundation for personalised strategies to improve cancer immunotherapy outcomes.

## 1. Introduction

Cancer immunotherapy has transformed the management of multiple malignancies by enabling the host immune system to recognise and eliminate tumour cells. Immune checkpoint inhibitors (ICIs) targeting programmed death-1/programmed death-ligand 1 (PD-1/PD-L1) and cytotoxic T-lymphocyte-associated antigen-4 (CTLA-4) have produced substantial clinical benefits across melanoma, non–small cell lung cancer (NSCLC), renal cell carcinoma (R’CC), hepatocellular carcinoma (HCC), urothelial carcinoma (UC), and subsets of colorectal cancer (CRC) [1,2,3,4,5]. Nevertheless, durable responses occur only in a fraction of patients because of primary or acquired resistance, while some develop clinically significant immune-related adverse events (irAEs) [6,7]. Identifying determinants of ICI efficacy and toxicity is therefore a central objective of precision oncology.

Early biomarker research emphasised tumour-intrinsic characteristics, including mutational burden, neoantigen load, and immune infiltration. Host-associated factors are now also recognised as important, and the gut microbiota has emerged as a potential regulator of systemic immunity and anti-tumour responses. The gastrointestinal ecosystem compromises bacteria, archaea, viruses, fungi, and their genetic elements that collectively support nutrient metabolism, epithelial barrier integrity, immune maturation, and inflammatory homeostasis [8]. Through interactions with innate and adaptive immune pathways, the microbiota can modulate dendritic cell maturation, antigen presentation, cytokine signalling, and T-cell priming, which contribute to effective ICI activity [9,10,11,12]. Dysbiosis has been associated with impaired ICI efficacy and shorter survival, particularly following antibiotic exposure [9,13,14]. These associations do not, however, establish causality: antibiotic use may also indicate infection, frailty, hospitalisation, disease severity, or other clinical factors associated with poorer outcomes.

Initial microbiome–immunotherapy studies have identified bacterial taxa such as *Akkermansia muciniphila*, *Bifidobacterium* spp., *Faecalibacterium prausnitzii*, and members of the Ruminococcaceae family to be enriched in responders [9,10,11]. Their reproducibility has been limited by differences in sequencing platforms, geographic regions, dietary patterns, treatment, bioinformatic pipelines, and response definitions [15,16]. No individual taxon has consistently predicted benefit across independent cohorts. Ecosystem-level features may be more informative: pathological complete response in triple-negative breast cancer was associated with greater microbial diversity, whereas *B. longum* represented only one component of the responder-associated community [17]. Conversely, self-administered probiotics were associated with lower diversity and poorer outcomes in patients with melanoma receiving anti-PD-1 therapy [18]. These observations suggest that microbial function and ecological structure may be more relevant than the abundance of any single organism.

Accordingly, the field is moving from taxonomic correlation towards mechanistic analysis of microbiome–immune interactions. Microbial metabolites, including short-chain fatty acids (SCFAs), secondary bile acids, inosine and tryptophan derivatives, can affect immune regulation and treatment outcomes [19,20,21,22,23]. Strain-level variation, ecological interactions, bacteriophage dynamics, and host-specific immune context may also modify responses to immunotherapy. Prophage-mediated adaptation in *Bacteroides fragilis*, for example, shows how strains within the same species can differ functionally and supports the use of strain-resolved analyses [24].

Because the microbiome is potentially modifiable, dietary strategies, prebiotics, probiotics, live biotherapeutic products (LBPs), faecal microbiota transplantation (FMT), postbiotics, and engineered microbial therapeutics are being investigated.

Here, a precision microbiome intervention means selecting a microbiome-directed strategy according to the patient’s baseline microbial ecology and function, host and tumour characteristics, and treatment history and the biological deficit that the intervention is intended to correct.

The distinctive contribution of this review is to translate heterogeneous microbiome findings into explicit criteria for clinical development. We distinguish association from causality, taxonomic prediction from functional mechanism, and biological modulation from clinical benefit, and use these distinctions to define requirements for biomarker validation, identification of modifiable biological deficits, mechanism-matched intervention selection and demonstration of microbial target engagement. This framework provides a consistent basis for comparing evidence across tumour types and determining when microbiome-guided strategies are ready for prospective clinical evaluation.

## 2. The Gut Microbiome as a Determinant of Immunotherapy Response

Mechanistic studies indicate that the microbiome influences anti-tumour immunity through interconnected metabolic, barrier, inflammatory, and immune pathways. These mechanisms provide a biological basis for the clinical associations between microbiome composition and ICI outcomes, while also explaining why taxonomic associations alone may be insufficient to predict therapeutic response. Figure 1 summarises these interconnected relationships. Importantly, microbiome restoration may improve immune competence but cannot necessarily overcome tumour-intrinsic mechanisms of ICI resistance.

### 2.1. From Taxonomic Associations to Functional Biomarkers

Early studies in melanoma and other solid tumours first demonstrated that gut microbial composition correlates with response to ICIs [9,10,11]. Transferring faecal microbiota from responding patients to germ-free or antibiotic-treated mice restored sensitivity to checkpoint blockade, providing early causal evidence. Subsequent studies, however, have not identified a consistently predictive individual taxon. Across melanoma and other solid-tumour cohorts, responders instead show different community configurations, often characterised by greater alpha diversity and separable beta-diversity profiles [15,16]. Species-level associations have important limitations. Detection of an organism does not demonstrate metabolic activity, ecological interaction, or immunological function, and strain-level variation can substantially alter host effects. Studies of *B. fragilis* in CRC illustrate how prophage-mediated adaptation and strain-specific virulence can modify host interactions [24]. Functional biomarkers may therefore be more informative. Integrating metagenomics, metabolomics, transcriptomics, and immune profiling can identify microbial pathways and host–microbe interactions that are biologically closer to ICI response than taxonomy alone. Candidate mediators include SCFAs, secondary bile acids, inosine and tryptophan-derived compounds, which can affect dendritic-cell activation, T-cell differentiation, epithelial-barrier integrity, and inflammatory signalling [19,21,22].

No microbial signature is currently universally predictive. ICI responsiveness appears to emerge from interactions among microbial ecology, metabolic output, host genetics, environmental exposures, and tumour-specific immune context. Human evidence remains constrained by small observational cohorts, inconsistent sampling, and response definitions, heterogeneous sequencing, and bioinformatic methods, and confounding by diet, medication exposure, geography, and disease severity. These limitations explain why responder-associated taxonomic signatures often fail to reproduce and why causal interpretation requires mechanistic or interventional confirmation.

### 2.2. Microbial Metabolites as Immune Modulators

Microbial metabolites are plausible functional mediators of ICI response because they link between microbial ecology and host immunity [19,20,21,22,23]. Their production and biological availability depend not only by taxonomic composition but also on strain-specific biosynthetic capacity, substrate availability, microbial cross-feeding, and host metabolism. Inosine produced by *B. pseudolongum* can promote Th1 differentiation through A2A receptor signalling [21], whereas tryptophan derivatives and secondary bile acids can alter immune tone through pathways including aryl hydrocarbon receptor signalling and the gut–liver axis [22,23]. Metabolite-based biomarkers must therefore account for biosynthetic potential, measured concentration, anatomical compartment, receptor availability, diet, concomitant medications, and treatment regimen. Within this broader metabolic network, the relationship between SCFAs and anti-tumour immunity is especially context dependent and warrants separate analysis.

#### Short-Chain Fatty Acids at the Tumour–Immune Interface: Context-Dependent Effects in Cancer

The association between SCFA-producing taxa and favourable immunotherapy outcomes does not imply that greater SCFA exposure is uniformly beneficial. Current evidence instead supports a conditional model in which acetate, propionate, butyrate, and pentanoate may act as metabolic substrates, signal through free fatty acid receptors 2 and 3 (FFAR2 and FFAR3), or alter transcription through histone deacetylase (HDAC) inhibition, with the biological outcome determined by the individual metabolite, concentration, anatomical compartment, exposed cell population, tumour metabolic phenotype, and treatment mechanism [19,25,26]. Compartment is particularly important: faecal concentrations represent the residual intestinal pool after microbial utilisation and epithelial absorption, whereas serum or plasma provides an indirect estimate of systemic exposure, urinary concentrations primarily reflect excretion, and tumour-tissue measurements are required to establish local availability. Butyrate is extensively consumed by colonocytes, while acetate and propionate undergo further hepatic and peripheral utilisation [25,26]. Accordingly, faecal, circulating, urinary, and intratumoral SCFAs are not interchangeable biomarkers. Recent NSCLC studies directly detected acetate enrichment in tumour tissue [27,28], illustrating why intestinal or circulating measurements cannot be assumed to reproduce metabolite exposure within the tumour microenvironment.

Acetate illustrates the competition between immune and malignant cells for the same metabolic resource. Under glucose restriction, acetate provided CD8^+^ T cells with an alternative source of acetyl-CoA through acetyl-CoA synthetase activity, increasing histone acetylation and restoring IFN-γ transcription and effector function. Suppression of this pathway impaired cytokine production by tumour-infiltrating lymphocytes and compromised tumour clearance [29]. Glucose restriction in this model represents nutrient competition within the tumour microenvironment and should not be interpreted as evidence that dietary fasting would reproduce the same effect. In breast-cancer models, inhibition of tumour-cell acyl-CoA synthetase short-chain family member 2 (ACSS2) changed malignant cells from acetate consumers to net producers, increased extracellular acetate available to infiltrating lymphocytes, and improved both T-cell activity and chemotherapy efficacy [30]. Thus, the relevance of acetate depends not only on its measured abundance but also on the relative capacities of tumour and immune cells to transport and metabolise it.

Conversely, acetate can be appropriated by malignant and suppressive myeloid cells. In human NSCLC tissue, acetate was the most-abundant detected SCFA. Uptake through monocarboxylate transporter 1 (MCT1) and conversion to acetyl-CoA by ACSS2 stabilised c-Myc, increasing PD-L1 expression, glycolytic and cell-cycle programmes, and acetate-transport machinery. Acetate supplementation promoted tumour growth and reduced CD8^+^ T-cell infiltration, whereas disruption of acetate uptake or metabolism enhanced anti-PD-1 efficacy [27]. A complementary study detected acetate accumulation in human lung-adenocarcinoma tissue and mouse tumours and identified FFAR2 expression on tumour-associated myeloid-derived suppressor cells (MDSCs). FFAR2 activation induced arginase 1 (ARG1), depleted L-arginine, and impaired T-cell activity; systemic or myeloid-specific deletion of *Ffar2* reduced MDSC accumulation, increased CD8^+^ infiltration, and improved immune-checkpoint-blockade efficacy [28]. These studies identify three competing acetate-dependent outcomes: metabolic rescue of nutrient-restricted cytotoxic T cells, metabolic support of malignant cells, and receptor-mediated activation of immunosuppressive MDSCs.

Butyrate can support antitumour immunity through metabolic, epigenetic, and receptor-dependent mechanisms. In germ-free mice, butyrate promoted oxidative phosphorylation, sustained glutamine utilisation, and fatty-acid catabolism in antigen-activated CD8^+^ T cells, improving memory formation and recall responses [20]. Although this was not a cancer model, it provides mechanistic evidence that a microbial metabolite can influence the persistence of antigen-experienced T cells. In MC38 colorectal adenocarcinoma, EG7 lymphoma, and colitis-associated colorectal-cancer models, butyrate enhanced oxaliplatin efficacy through interleukin-12 (IL-12) signalling and inhibitor of DNA binding 2 (ID2)-dependent cytotoxic CD8^+^ T-cell activity. Butyrate also enhanced anti-PD-L1 efficacy in the MC38 model. In an accompanying observational cohort of patients with gastrointestinal cancers receiving oxaliplatin, responders had higher naturally occurring serum butyrate concentrations than non-responders. However, this clinical association was specific to chemotherapy response and did not evaluate immunotherapy. Thus, although the anti-PD-L1 experiment provides preclinical evidence relevant to ICI therapy, the human data do not establish butyrate as a clinical biomarker of immunotherapy response [22].

Controlled exposure may also be relevant to cellular immunotherapy. Ex vivo conditioning with pentanoate or butyrate increased CD25, IFN-γ, and TNF-α in antigen-specific cytotoxic T lymphocytes and chimeric antigen receptor T (CAR-T) cells through mechanistic target of rapamycin (mTOR) activation and class I HDAC inhibition. Adoptive transfer of conditioned cells improved tumour control in B16-OVA melanoma and PancOVA pancreatic cancer models [31]. This finding supports temporally controlled metabolic programming of therapeutic lymphocytes more directly than unrestricted systemic supplementation, which would simultaneously expose tumour cells, regulatory T cells, and suppressive myeloid populations.

Several studies further connect butyrate with PD-1-based therapy. Administration of *Roseburia intestinalis* or butyrate increased granzyme B^+^, IFN-γ^+^, and TNF-α^+^ CD8^+^ T cells and improved anti-PD-1 activity in colorectal-cancer models. The investigators proposed direct activation of Toll-like receptor 5 (TLR5)-nuclear factor kappa B (NF-κB) signalling in CD8^+^ T cells, although TLR5 is not conventionally considered a principal SCFA receptor and this mechanism requires independent confirmation [32]. In a cohort of 22 patients with NSCLC receiving anti-PD-1 combined with chemotherapy, responders had higher circulating acetate, propionate, and butyrate. Mechanistic experiments showed that butyrate increased acetylation of histone H3 at lysine 27 (H3K27ac) at the *PDCD1* and *CD28* promoters, strengthening T-cell-receptor signalling; in melanoma-bearing mice, butyrate enhanced anti-PD-1 efficacy through a CD8^+^ T-cell-dependent mechanism [33]. The small, combination-treatment cohort nevertheless limits attribution of the human association specifically to PD-1 blockade. In gastric cancer, butyrate reduced PD-L1 and IL-10 in patient-derived immune cells and inhibited tumour growth in an avatar model, suggesting that its effects can extend to tumour-associated macrophages; this remained a patient-derived experimental model rather than a clinical intervention [34].

The same metabolites can produce the opposite effect when systemic exposure alters antigen presentation or regulatory-cell balance. In metastatic melanoma, high circulating butyrate and propionate were associated with reduced benefit from the anti-CTLA-4 antibody ipilimumab and with increased regulatory T-cell proportions. In mouse models, oral sodium butyrate limited dendritic-cell maturation, reduced CD80/CD86 upregulation, and impaired the accumulation of tumour-specific and memory T cells during CTLA-4 blockade [19]. In vitro, butyrate and propionate suppressed IL-12 and IL-23 production by human monocyte-derived dendritic cells and impaired antigen-specific CD8^+^ T-cell activation; IL-12 supplementation restored activation [35]. These findings help reconcile apparently contradictory results: SCFAs may improve the metabolism and persistence of an already activated cytotoxic T cell while impairing the dendritic-cell functions required to prime a new response. They also demonstrate that an SCFA profile associated with benefit during PD-1-based treatment cannot automatically be considered favourable during CTLA-4 blockade.

Direct effects on malignant cells add a further layer of context dependence. In colorectal-cancer cells exhibiting the Warburg phenotype, inefficient oxidation allowed butyrate to accumulate, inhibit HDAC activity, and suppress proliferation. When glycolysis was experimentally reduced, the same cells oxidised butyrate as a carbon and energy source, and a low concentration promoted rather than inhibited proliferation [36]. Butyrate and propionate can also increase colorectal-cancer immunogenicity: in cell lines, patient-derived organoids and orthotopic models, they induced HDAC-dependent DNA-damage responses, chemokines, major histocompatibility complex class I, and antigen-presentation pathways, thereby promoting CD8^+^ T-cell activation; microsatellite-unstable models were more sensitive than microsatellite-stable models [37]. The contrast between enhanced antigen-presentation programmes in malignant cells [37] and impaired antigen presentation by dendritic cells [35] demonstrates that the identity of the responding cell can reverse the net immune consequence of the same SCFA.

Tumour-promoting effects have also been demonstrated in prostate cancer. A 1:1 acetate–butyrate mixture induced tumour-cell autophagy, Toll-like receptor 3 (TLR3), NF-κB and mitogen-activated protein kinase (MAPK) signalling, and C-C motif chemokine ligand 20 (CCL20) release in prostate-cancer models. CCL20 promoted macrophage recruitment and M2 polarisation, generating a tumour-supportive microenvironment and increasing migration and invasion; because the metabolites were administered together, their individual contributions could not be separated [38]. In prostate-specific *Pten*-deficient mice, high-fat-diet-associated microbiome changes increased SCFA availability, insulin-like growth factor 1 (IGF1), and downstream MAPK/phosphoinositide 3-kinase signalling, promoting tumour proliferation. The accompanying human observations did not establish SCFA-mediated causality in patients [39].

Collectively, these data do not support classifying SCFAs as uniformly antitumour or tumour-promoting. Their net effect emerges from competition among tumour, lymphoid, and myeloid cells and varies with metabolite identity and dose, cellular activation state, tumour metabolic phenotype, anatomical compartment, and therapy [40]. Acetate may preserve CD8^+^ T-cell function under nutrient restriction but also sustain c-Myc/PD-L1 signalling or FFAR2/ARG1-mediated suppression [27,28,29,30]. Butyrate can enhance T-cell memory, cellular immunotherapy, chemotherapy, and PD-1-based treatment, yet butyrate and propionate may suppress dendritic-cell priming and reduce CTLA-4-blockade efficacy [19,20,22,31,32,33,34,35]. Direct tumour-cell effects range from HDAC-mediated growth inhibition and enhanced immunogenicity in colorectal cancer to macrophage- and IGF1-dependent tumour promotion in prostate cancer [36,37,38,39].

This context dependence has direct implications for biomarker development and intervention design. Enrichment of SCFA-producing bacteria or elevation of faecal SCFAs should not be interpreted as evidence of favourable intratumoral activity. Future studies should combine longitudinal microbiome profiling with targeted quantification of individual SCFAs in matched faecal, serum, or plasma and tumour-tissue samples, with urinary metabolomics used as a complementary measure of excretion rather than a surrogate for tumour exposure. These data should be integrated with tumour-cell metabolic phenotype, transporter, enzyme and receptor expression, spatial immune profiling, treatment mechanism, and longitudinal clinical response. Intervention studies should demonstrate both modulation of the intended SCFA pathway and the predicted immune effect within tumour tissue before clinical benefit is attributed to dietary, microbial, or metabolite-based strategies.

### 2.3. Barrier Integrity and Inflammatory Signalling

Beyond microbial metabolite production, maintenance of intestinal barrier integrity represents a critical mechanism linking the gut microbiome to systemic immune regulation. Dysbiosis can compromise intestinal epithelial barrier integrity, resulting in increased permeability and systemic translocation of microbial products, including lipopolysaccharide (LPS). Disruption of the epithelial barrier reduces the spatial separation between commensal microbes and host immune cells, allowing microbial products to activate pattern recognition receptors (PRRs), including Toll-like receptor 4 (TLR4), on epithelial and immune cells. Persistent LPS–TLR4 signalling promotes NF-κB activation and sustained production of pro-inflammatory cytokines, contributing to chronic inflammation and immune dysregulation [8]. In the context of cancer, chronic inflammation and immune dysregulation have been associated with the development of immune-excluded or “cold” tumour microenvironments characterised by reduced cytotoxic T-cell infiltration and diminished responsiveness to immune checkpoint inhibitors [41]. Thus, disruption of barrier integrity may represent an important mechanistic link between gut dysbiosis, microbial translocation, chronic inflammatory signalling, and immunotherapy resistance.

### 2.4. Host and Tumour Factors Shaping Microbiome–Immune Interactions

Microbiome-associated immunotherapy responses are further influenced by host-specific and tumour-intrinsic factors that modulate both microbial ecology and anti-tumour immunity. Host genetic variation contributes to interindividual differences in microbial composition [42] while HLA class I genotype can independently influence response to checkpoint blockade [43]. Tumour-intrinsic alterations can also sustain immune exclusion or primary resistance independently of the microbiome. WNT/β-catenin activation can restrict T-cell infiltration [41], *JAK1/2* defects can impair interferon signalling [44], and *STK11/LKB1* alterations are associated with resistance in *KRAS*-mutant lung adenocarcinoma [45]. These variables should therefore be analysed alongside microbiome features rather than attributed to them. For precision stratification, host genetic and tumour-intrinsic variables should be analysed alongside, rather than attributed to, the microbiome.

### 2.5. The Virome and Mycobiome: Neglected Functional Layers

Bacteriophages can restructure bacterial communities through predation, lysogeny, horizontal gene transfer, and modulation of bacterial fitness [46]. The *B. fragilis* prophage findings illustrate how viral carriage may alter strain behaviour even when bacterial species abundance appears unchanged [24]. FMT can transfer donor bacteriophages together with bacteria [47]; however, most oncology trials do not quantify the virome, making it impossible to determine whether bacterial engraftment is causal or instead marks broader ecosystem transfer. Fungi can influence local and systemic immunity through pattern-recognition pathways, but direct, reproducible evidence linking a defined gut mycobiome state to ICI outcome remains limited [48]. Future trials should therefore include virus-like-particle or total metagenomic sequencing, fungal profiling, and longitudinal strain/phage tracking. These data should initially be treated as exploratory functional layers rather than clinically actionable biomarkers.

### 2.6. Clinical Evidence for Microbiome–Immunotherapy Interactions

Across multiple cancers, antibiotic exposure before or during ICI therapy is associated with reduced progression-free survival (PFS) and overall survival (OS) [13,14]. Although confounding factors exist, these findings consistently suggest that microbiome disruption impairs therapeutic efficacy. Mechanistically, antibiotic-induced depletion of immunostimulatory microbial communities may reduce the microbial signals and metabolites required for effective systemic antitumour immunity [9,10,11,12]. Microbial composition has also been linked to irAEs, particularly checkpoint inhibitor–associated colitis [16,49]. In patients with metastatic melanoma receiving ipilimumab, a baseline microbiota enriched in *Faecalibacterium* and other Firmicutes was associated with longer PFS and a higher incidence of ipilimumab-induced colitis, suggesting that microbial features associated with enhanced antitumour immunity may also predispose to intestinal immune toxicity [49]. Similarly, in patients with melanoma receiving anti-PD-1 therapy, microbial signatures enriched in Lachnospiraceae and Streptococcaceae were associated with distinct patterns of irAEs, highlighting that the relationship between microbial composition and toxicity may depend on the specific immune checkpoint pathway being targeted [16]. Together, these observations support the microbiome as a candidate modifier of ICI efficacy and toxicity, but not as a stand-alone determinant.

### 2.7. Translational Implications for Integrated Biomarker Development

Taken together, these mechanistic and clinical observations provide a biological basis for microbiome biomarker development. However, no universally applicable microbial biomarker has yet entered routine clinical practice. Responder-associated signatures frequently fail to reproduce because microbial functions vary according to strain-level properties, ecological context, diet, medication exposure, geography, host physiology, and tumour characteristics [15,16]. Consequently, taxonomic composition alone provides only a partial representation of the biological processes influencing antitumour immunity.

A precision framework must answer three separate questions: Is there a reproducible biological deficit? Is that deficit plausibly modifiable? Can the selected intervention correct it safely? Taxonomy alone answers none of them. A candidate biomarker panel should combine microbial ecology and functional pathways with faecal or circulating metabolites, inflammatory markers, immune-cell phenotypes, tumour genomics, treatment history, diet, and microbiome-disrupting medication. Any predictive model should be locked before validation, calibrated across sites, and tested for incremental value beyond established oncology biomarkers.

This approach shifts the emphasis from descriptive taxonomic profiling to integrated functional models of microbial metabolism, immune regulation, and host–microbe interaction. Studies of inosine and SCFAs demonstrate that microbial activity can influence antitumour immunity [20,21,22], but they also show that the effect depends on biological context. Patient stratification and intervention selection will therefore require combined microbial, metabolic, immune, and clinical data rather than reliance on individual taxa.

### 2.8. A Provisional Precision-Microbiome Decision Framework

Building on the conceptual model above, the principal translational challenge is to determine how microbiome, host, and tumour information could support patient stratification and intervention selection. We therefore propose a provisional, hypothesis-generating research framework rather than a validated clinical algorithm or current standard of care. First, tumour eligibility for ICI should be established using tumour-specific and clinical criteria, while tumour-intrinsic resistance mechanisms unlikely to be corrected by microbiome modulation should also be considered [7,41,43,44,45]. Second, the baseline host–microbiome state should be characterised using standardised metagenomic analysis, targeted metabolomics, inflammatory and immune profiling, medication and antibiotic exposure, dietary fibre intake, and, where feasible, HLA or other host-genetic features [13,14,15,16,18,42,43]. Third, the dominant potentially modifiable biological deficit should be identified rather than assigning patients to responder or non-responder categories based solely on taxonomic profiles, which have limited reproducibility across cohorts [15,16]. The intervention evaluated should be matched to the identified deficit. Patients with low fibre intake but otherwise preserved microbial ecology could be evaluated prospectively in supervised fibre-rich dietary studies with longitudinal metabolic and immunological monitoring; this proposal is supported by the association between higher fibre intake and improved ICI outcomes in melanoma but remains to be validated as an intervention strategy [18]. Patients with marked dysbiosis or depletion of a defined microbial function could instead be considered for trials of a mechanism-matched LBP or defined consortium [50,51,52,53,54]. Antibiotic-associated loss of diversity alone should not be treated as evidence that an LBP will reverse ICI resistance, because the available antibiotic studies are observational [13,14]. FMT should remain restricted to clinical trials or appropriately regulated settings: early melanoma studies demonstrate engraftment and restored anti-PD-1 sensitivity in a subset of patients [55,56], while donor effects, pathogen transmission, and long-term durability remain important uncertainties [57,58]. Finally, biological mechanism engagement should be demonstrated before clinical efficacy is inferred. Evaluation should include donor or therapeutic-strain engraftment, restoration of the targeted microbial pathway, relevant metabolite changes, and the predicted immune effect, followed by prospective assessment of radiological response, progression-free survival, toxicity, and quality of life [50,52,53,54,55,56]. This staged design distinguishes biological modulation from clinical benefit and may help attribute non-response to failed delivery, absent engraftment, lack of functional restoration, insufficient immune modulation, or persistent tumour-intrinsic resistance. The next section applies this framework across tumour types, asking not merely which taxa were reported, but whether the evidence is reproducible, mechanistically plausible, and sufficiently mature to inform clinical development.

## 3. Clinical and Translational Evidence Across Solid Tumours

Clinical studies across solid tumours increasingly support a role for the gut microbiome as a context-dependent modifier of ICI efficacy. However, the strength, consistency, and mechanistic plausibility of these associations vary markedly between cancer types. Differences in tumour immunogenicity, baseline inflammation, comorbidities, and exposure to antibiotics or other microbiome-altering medications shape the extent to which microbial ecosystems influence therapeutic outcomes. This section gathers tumour-specific evidence, emphasizing clinical observations and translational findings rather than mechanisms already discussed in earlier sections.

### 3.1. Melanoma

Melanoma remains the most robust clinical model linking the gut microbiome to ICI outcomes [9,10,11]. It is also the tumour type in which microbiome-directed therapeutic strategies have progressed furthest into clinical development. Initial proof-of-concept phase I studies demonstrated that responder-derived FMT combined with PD-1 blockade could restore clinical responses and achieve durable disease control in a subset of PD-1-refractory patients [55,56]. Subsequent studies have expanded this approach to other donor and treatment settings. The phase I MIMic study evaluated healthy-donor FMT combined with first-line anti-PD-1 therapy [59], while the phase 2a PICASSO trial investigated the pooled-donor microbiota product MaaT013 in combination with ipilimumab and nivolumab (NCT04988841). Defined LPB have also entered clinical evaluation, including EDP1503 (*B. animalis* subsp. *lactis*; NCT03775850) and SER-401 combined with nivolumab (NCT03817125) [52,54]. Collectively, these studies position melanoma as the leading clinical model for evaluating microbiome-guided immunotherapy, although larger randomised trials are still required to establish efficacy and identify robust predictive biomarkers.

Despite these advances, reproducibility of specific microbial biomarkers remains limited across cohorts, reflecting differences in diet, geography, sequencing methods, and baseline immune status. Nevertheless, melanoma provides the clearest proof-of-concept that the microbiome can function as both a predictive biomarker and a therapeutic target.

### 3.2. Colorectal Cancer (CRC)

CRC is strongly shaped by intestinal microbial ecology, yet the clinical relevance of the microbiome for ICI efficacy is highly dependent on tumour genomics. Patients with mismatch repair-deficient/microsatellite instability-high (dMMR/MSI-H) tumours respond well to PD-1 blockade [60,61], whereas microsatellite-stable (MSS) CRC, which represents the majority of cases, remains largely refractory to immunotherapy. Microbial signatures in CRC primarily reflect tumour biology and carcinogenesis rather than ICI responsiveness. Nevertheless, some carcinogenesis-associated microorganisms may also shape the tumour immune contexture. For example, *Fusobacterium nucleatum* and colibactin-producing *Escherichia coli* have been associated with an immunosuppressive tumour microenvironment as well as colorectal carcinogenesis [62,63,64]. These observations have prompted the development of several microbiome-directed therapeutic strategies in CRC. Ongoing clinical trials are currently evaluating responder-derived FMT combined with PD-1 blockade (NCT05250635), live microbial consortia (NCT03686202), dietary modulation with pectin supplementation (NCT03704272), and established immunotherapy regimens in patients with MSI-H/dMMR disease, including nivolumab (CheckMate-142; NCT02060188) and pembrolizumab (NCT01876511). Although these approaches are promising, clinical evidence remains preliminary, particularly for patients with MSS CRC.

At present, the microbiome in CRC appears more relevant as a driver of tumourigenesis and immune contexture than as a validated predictor of ICI response.

### 3.3. Non–Small Cell Lung Cancer (NSCLC)

In NSCLC, clinical evidence supports a modulatory role for the gut microbiome, though findings are less consistent than in melanoma. The most reproducible observation is the negative impact of recent antibiotic exposure, which correlates with reduced response rates and shorter survival [13]. Among individual taxa, *A. muciniphila* has emerged as one of the most consistently reported microbial correlates of favourable outcomes [65]. Interpretation is complicated by confounders common in NSCLC, including smoking [66], chronic obstructive pulmonary disease (COPD) [67], chronic infections [8,68], proton pump inhibitor use [69], and prior systemic therapies [70,71], all of which independently influence microbial composition and systemic inflammation. Retrospective clinical studies have also suggested that the live biotherapeutic product *Clostridium butyricum* MIYAIRI 588 (CBM588) [71] may improve clinical outcomes in patients receiving chemoimmunotherapy, although prospective validation is still required [72]. More recently, the phase II FMT-LUMINate study evaluated healthy-donor FMT combined with first-line ICI therapy in patients with NSCLC and melanoma. In the NSCLC cohort, responders developed distinct post-FMT microbial changes, characterised by greater loss of baseline bacterial species rather than consistent donor-strain engraftment [73]. Although encouraging, these findings require confirmation in larger randomised controlled studies capable of distinguishing the contribution of FMT from that of ICI therapy.

### 3.4. Hepatocellular Carcinoma (HCC)

HCC develops within a distinctive immunometabolic environment shaped by chronic liver disease and continuous exposure to gut-derived microbial products. Gut microbiome and metabolite profiles have been associated with ICI outcomes in unresectable HCC, although cirrhosis-related barrier dysfunction and microbial translocation represent important potential confounders [74]. A distinctive mechanistic feature is the bile acid-dependent gut–liver immune axis, through which microbial regulation of bile acid composition can influence hepatic natural killer T-cell recruitment and anti-tumour immunity [23]. However, cirrhosis, portal hypertension, and aetiology-specific inflammation associated with viral hepatitis, metabolic dysfunction-associated steatotic liver disease (MASLD)/NASH, and alcohol-related liver disease may independently affect both microbial ecology and host immunity, making microbiome-specific effects difficult to isolate.

Microbiome-targeted interventions in HCC remain investigational. The multicentre, randomised, placebo-controlled phase II FLORA trial (NCT05690048) is evaluating FMT combined with atezolizumab–bevacizumab in advanced HCC; efficacy results are not yet available [75]. In addition, a randomised dietary intervention incorporating probiotics, prebiotics and a high-fibre diet reported modulation of the gut microbiome together with improvements in selected clinical outcomes [76]. A separate clinical study (NCT05032014) is evaluating *Lactobacillus rhamnosus* Probio-M9 supplementation in combination with PD-1 therapy in liver cancer. Although these approaches indicate increasing clinical interest in microbiome modulation, the evidence remains preliminary and requires confirmation in adequately powered prospective randomised studies.

### 3.5. Renal Cell Carcinoma (RCC)

RCC is one of the tumour types with the most consistent observational evidence linking microbial composition to ICI outcomes. Responders frequently show enrichment of *A. muciniphila*, members of the Ruminococcaceae, and other taxa associated with metabolically active gut communities [77]. The most compelling early clinical evidence for microbiome modulation has also emerged in RCC. In a randomised phase I study, *C. butyricum* MIYAIRI 588 (CBM588) combined with nivolumab–ipilimumab improved progression-free survival and increased beneficial gut microbial taxa [50]. More recently, the PERFORM phase I and TACITO phase 2a studies demonstrated the feasibility of responder-derived FMT as an adjunct to ICI therapy, with encouraging engraftment and preliminary clinical activity [78,79]. Additional studies continue to evaluate FMT with ICIs (e.g., NCT05273255). RCC may be particularly responsive to microbiome modulation because its immunogenic biology and reliance on systemic T-cell priming create a plausible route for gut-derived immune effects. Nevertheless, these trials are small and no microbial biomarker is ready for routine clinical use; larger prospective randomised studies are required.

### 3.6. Urothelial and Bladder Cancer

Evidence concerning microbiome–immunotherapy interactions in urothelial carcinoma (UC) and bladder cancer remains less mature than that available for melanoma, non-small-cell lung cancer (NSCLC) or renal cell carcinoma (RCC). Current evidence has focused primarily on the urinary microbiome, a low-biomass but potentially immunologically active niche that may influence tumour biology, local immune responses, and treatment outcomes [80,81,82,83]. Available studies suggest that patients with bladder cancer exhibit alterations in urinary microbial diversity and community composition. However, findings vary substantially because of small sample sizes, heterogeneous sampling methods—including midstream urine, catheterised urine, and tumour tissue and methodological variability [82,83].

Given the established role of intravesical Bacillus Calmette–Guérin (BCG) in non-muscle-invasive bladder cancer, the urinary microbiome may influence local immune activation and therapeutic response, although direct evidence remains limited. Its relationship with systemic ICI efficacy is also largely unexplored.

Gut-microbiome evidence is beginning to emerge. In an exploratory stool analysis from the PURE-01 neoadjuvant pembrolizumab study, *Sutterella* was enriched in pathological responders, whereas *Ruminococcus bromii* was enriched in non-responders [84]. These preliminary associations require confirmation in independent cohorts before they can be considered reproducible biomarkers.

Importantly, urinary and intestinal microbiome signals represent distinct biological compartments and should not be conflated. The urinary microbiome may be particularly relevant to the local tumour environment and intravesical treatment, whereas the intestinal microbiome may influence systemic anti-tumour immunity. Future prospective studies should evaluate these compartments separately using standardised collection and analytical procedures while accounting for relevant clinical confounders. At present, neither urinary nor intestinal microbial signatures have been validated for selecting patients for systemic ICI therapy, and the evidence remains hypothesis-generating.

### 3.7. Cross-Tumour Synthesis: Why the Evidence Differs

The apparent contribution of the microbiome to ICI response varies across tumour types because it is shaped by the underlying immunogenicity of each cancer, established tumour-intrinsic determinants of response, treatment regimens and disease-specific confounders. The evidence is most mature where observational findings have been complemented by mechanistic studies and early microbiome-directed interventions, as in melanoma and RCC [15,50,55,56,77,79]. In other settings, microbiome associations are more difficult to interpret because factors such as concomitant medications, organ dysfunction, environmental exposures, and differences among intestinal, urinary, and tumour-associated microbial compartments may independently affect both microbial profiles and clinical outcomes [13,23,65,74,81,82]. Moreover, established tumour biomarkers, particularly dMMR/MSI-H status in CRC, may have substantially greater predictive value than currently reported microbiome signatures [5,60,61]. These differences indicate that microbiome biomarkers are unlikely to be universally transferable across cancers and should instead be validated within specific tumour, treatment, and clinical contexts. The current strength of the evidence, principal findings, and limitations across tumour types are compared in Table 1. Across tumour types, the most reproducible responder-associated features are ecological and functional rather than taxonomic. Higher microbial diversity, enrichment of SCFA-producing taxa such as *Faecalibacterium* and members of the Ruminococcaceae, and reduced dominance of Proteobacteria and pathobionts recur across several melanoma and ICI cohorts, although none represents a universal signature [9,10,11,15,16]. By contrast, several signals appear context specific: *A. muciniphila* has been most consistently associated with outcomes in NSCLC and RCC [65,77], bile-acid-related microbial features are particularly relevant in HCC [23,74], and findings in urothelial cancer differ depending on whether urine, tumour tissue, or stool is examined [80,81,82,83,84]. Thus, the most conserved signals currently reside at the level of ecosystem structure and function, whereas individual taxa require tumour-, treatment- and compartment-specific validation. From a clinical perspective, these comparisons indicate different levels of evidence and translational readiness across tumour types. Melanoma currently provides the strongest evidence, supported by independent responder-associated cohorts, cross-cohort validation, and early responder-derived FMT studies [9,10,11,15,16,55,56]. RCC has emerging interventional evidence from CBM588 supplementation and responder-derived FMT, although the available trials remain small [50,77,78,79]. Evidence in NSCLC remains predominantly observational, with antibiotic exposure and *A. muciniphila* representing the most consistently reported associations [13,65,73]. By comparison, microbiome findings in HCC remain closely linked to the gut–liver axis and underlying liver dysfunction [23,74,75,76], while evidence in urothelial cancer remains exploratory and dependent on the microbial compartment analysed [80,81,82,83,84]. In CRC, microbial features are currently more strongly associated with carcinogenesis and tumour immune regulation than with prediction of ICI response, for which dMMR/MSI-H status remains the established biomarker [5,60,61,62,63,64]. Therefore, microbiome profiles should not yet be used as stand-alone clinical tests, and any proposed signature requires prospective validation within its specific tumour, treatment, and anatomical context.

## 4. Dietary and Therapeutic Interventions

### 4.1. Dietary Modulation and Nutritional Interventions

Diet is a strong and rapidly modifiable determinant of gut microbial composition and function. Although host genetics contributes to microbiome variation [42], controlled feeding studies show that dietary changes can alter microbial community structure within days [85]. Nutritional interventions are therefore attractive candidates for prospective evaluation alongside cancer immunotherapy, but association should not be interpreted as proof of therapeutic benefit.

Among dietary factors, fibre has received particular attention because it supports microbial diversity and provides substrates for bacterial fermentation, including members of the Ruminococcaceae and Lachnospiraceae families [86]. Nevertheless, the relationship between fibre intake, SCFA production, and antitumour immunity is not linear, as the biological effects of individual SCFAs depend on their concentration, anatomical compartment, cellular target, tumour metabolic phenotype, and treatment context, as discussed in detail above [19,20,22,25,26,27,28,29,30,31,32,33,34,35,36,37,38,39,40].

Spencer et al. reported that melanoma patients consuming a high-fibre diet experiencing significantly improved PFS during anti-PD1 therapy compared to patients with lower dietary fibre intake [18].

Importantly, higher fibre consumption was associated with increased microbial diversity and enrichment of bacterial communities previously linked to favourable immunotherapy responses [18]. Moreover, each 5 g/day increase in dietary fibre intake was associated with an approximately 30% reduction in the risk of disease progression or death. In contrast, patients who self-administered commercially available probiotic supplements exhibited lower microbial diversity and a trend towards poorer clinical outcomes despite otherwise favourable dietary patterns. Complementary preclinical experiments further demonstrated that probiotic supplementation impaired anti–PD-1 efficacy, reduced intratumoural IFN-γ^+^ CD8^+^ T-cell responses and altered gut microbial composition [18]. Collectively, these findings highlight the complexity of microbiome modulation and suggest that promoting a diverse, fibre-supported microbial ecosystem may be more beneficial than indiscriminate probiotic supplementation during immune checkpoint inhibitor therapy.

Beyond fibre, broader dietary patterns may also influence immunotherapy outcomes through their effects on microbial ecology and metabolic function. Diets rich in fruits, vegetables, legumes, whole grains, and plant-derived polyphenols are generally associated with increased microbial diversity and enhanced production of immunomodulatory metabolites [87,88]. The resulting increase in microbial diversity and beneficial microbial metabolites may create a gut environment that supports more effective antitumour immune responses and enhances responsiveness to immune checkpoint blockade. Conversely, the global adoption of Western dietary patterns has been associated with reduced microbial diversity and functional capacity, potentially compromising microbiome-dependent immune regulation. These changes may have broader implications for cancer prevention and could reduce the effectiveness of microbiome-targeted therapeutic strategies across different populations, highlighting the importance of preserving healthy dietary patterns in precision oncology [89,90].

Despite strong biological plausibility, several limitations currently prevent the routine implementation of dietary interventions as adjuncts to immunotherapy. Most available evidence remains observational, dietary adherence is difficult to standardize, and microbiome responses vary considerably between individuals [85]. Furthermore, nutritional studies [88,89] are frequently confounded by lifestyle factors, obesity, medication use, and underlying disease status. Consequently, although dietary modulation represents one of the most accessible and safest microbiome-targeting strategies currently available, prospective randomised clinical trials are required to determine whether specific dietary interventions can reproducibly enhance immunotherapy efficacy.

Collectively, the current evidence suggests that diet influences immunotherapy outcomes primarily through modulation of microbial function rather than promotion of specific bacterial taxa. This observation reinforces the emerging concept that functional microbiome pathways may be more clinically relevant than taxonomic composition alone and supports the integration of nutritional strategies into future microbiome-guided precision oncology approaches.

### 4.2. Faecal Microbiota Transplantation (FMT)

Among currently available microbiome-modulating strategies, FMT has generated the strongest clinical evidence supporting microbiome-directed modulation of cancer immunotherapy outcomes. FMT involves the transfer of a complex microbial community from a healthy donor or selected responder into a recipient with the aim of restoring microbial diversity and function. Unlike probiotics or dietary interventions, which typically target individual taxa or metabolic pathways, FMT transfers an entire microbial ecosystem, including bacteria, archaea, viruses, bacteriophages, fungi, and their associated metabolic networks.

The rationale for FMT in oncology was established through seminal preclinical studies demonstrating that FMT from patients responding to immune checkpoint inhibitors (ICIs) could transfer sensitivity to PD-1 blockade when transplanted into germ-free or antibiotic-treated mice, providing causal evidence that the gut microbiome can influence immunotherapy efficacy [9,10,11]. Two landmark phase I studies published in 2021 subsequently established the clinical proof-of-concept that responder-derived FMT, combined with PD-1 blockade, can partially overcome acquired resistance to anti–PD-1 therapy in patients with metastatic melanoma [55,56]. Clinical benefit was associated with successful donor microbiome engraftment, enhanced CD8^+^ T-cell activation, increased antigen presentation, and upregulation of interferon-γ-related immune pathways, supporting the concept that microbiome modulation can restore responsiveness to immune checkpoint blockade.

Subsequent investigations have sought to identify the mechanisms underlying successful FMT responses. Successful donor microbiota are frequently enriched in *A. muciniphila*, *Faecalibacterium* spp., *Bifidobacterium* spp., and other taxa commonly associated with favourable immunotherapy outcomes [9,10,11]. However, growing evidence suggests that successful engraftment may depend less on individual taxa and more on restoration of microbial diversity, metabolic function, epithelial barrier integrity, and immune homeostasis. Consequently, attention has increasingly shifted toward donor functional profiles, metabolomic signatures, and ecological compatibility rather than taxonomic composition alone.

Despite encouraging results, several limitations currently restrict widespread implementation of FMT in oncology. Clinical responses remain donor-dependent, and not all responder-derived microbiota produce equivalent therapeutic effects. Standardization of donor selection, screening procedures, stool processing, administration routes, and long-term monitoring remains incomplete [57]. Furthermore, concerns regarding transmission of multidrug-resistant organisms, opportunistic pathogens, and unforeseen metabolic consequences have prompted increasing regulatory scrutiny [58]. These challenges are particularly relevant in immuno-compromised cancer populations, where safety considerations are paramount.

Another important limitation is the durability of engraftment. Long-term persistence of donor microbial communities varies substantially between recipients and may be influenced by diet, medication exposure, baseline microbiome composition, and host immune status [91]. Consequently, repeated administration or combination with adjunctive strategies such as dietary modification, prebiotics, or defined microbial therapeutics may be required to sustain clinical benefit.

Building on the encouraging melanoma results [55,56,59], FMT-based strategies are being evaluated across NSCLC, RCC, HCC, CRC, UC, and mixed solid tumours (Table 2). Across published cohorts, responder-associated profiles repeatedly include functionally diverse, SCFA-producing communities containing *Faecalibacterium*, *Eubacterium*, *Ruminococcus*, and *Bifidobacterium*, whereas resistance is often accompanied by low-diversity, Proteobacteria- or Enterobacteriaceae-rich configurations and poor donor integration [9,10,11,55,56,59,78]. These are recurring ecological patterns, not universal diagnostic signatures: the contributing taxa, treatment context, and evidence quality differ by study. Results are most mature in melanoma and RCC; most other tumour-specific FMT studies remain early phase or pending. Larger randomised trials must therefore define donor selection, schedule, mechanism engagement, patient stratification, and durability before routine use.

### 4.3. Live Biotherapeutic Products and Defined Microbial Consortia

FMT provides proof of concept, but donor variability, inconsistent engraftment, manufacturing complexity, and safety concerns limit routine implementation. These constraints have encouraged development of LBPs: defined microorganisms manufactured under controlled conditions to deliver a reproducible biological effect.

Unlike conventional probiotics, which are often selected based on historical use rather than mechanistic rationale, LBPs are designed to target specific pathways involved in host immunity, microbial metabolism, and tumour–microbiome interactions. This approach offers several potential advantages, including improved standardization, reduced batch-to-batch variability, enhanced regulatory oversight, and the possibility of identifying microbial mechanisms directly responsible for therapeutic efficacy.

One of the earliest clinical examples of an LBP is *C. butyricum* MIYAIRI 588 (CBM588), a butyrate-producing bacterium developed to modulate the gut microbiome and enhance host immune responses during ICI therapy. In a randomised phase I study in metastatic RCC, CBM588 administered in combination with nivolumab and ipilimumab demonstrated an acceptable safety profile together with preliminary biological and clinical activity, supporting the feasibility of defined microbial therapeutics in oncology [50]. Treatment was associated with remodelling of the gut microbiome, including enrichment of *Bifidobacterium* spp. and increased microbial diversity, suggesting that targeted microbial interventions may reshape broader microbial ecosystem structure rather than acting through a single bacterial species. Beyond single-strain approaches, increasing attention has focused on defined microbial consortia designed to reproduce the immunological and functional characteristics of responder-associated microbiomes. Tanoue et al. (2019) identified a consortium of eleven commensal bacterial strains capable of inducing interferon-γ-producing CD8^+^ T cells and enhancing antitumour immunity in preclinical models [51]. This work provided a mechanistic framework for the development of next-generation LBPs based on rationally selected microbial communities rather than individual bacterial strains. Several defined microbial therapeutics have subsequently entered clinical development with the aim of reproducing the beneficial immunomodulatory effects of responder-derived microbiota while overcoming the logistical and regulatory limitations of FMT. SER-401 was developed as a rationally designed consortium intended to restore beneficial microbial functions following antibiotic exposure with the aim of enhancing responsiveness to checkpoint blockade [52]. Similarly, VE800, a defined consortium of cultured human commensal bacteria, was specifically engineered to stimulate cytotoxic T-cell responses and improve immunotherapy efficacy [53]. EDP1503, an orally administered preparation containing *B. animalis* subsp. *lactis*, has also been evaluated in combination with pembrolizumab in patients with advanced solid tumours. Phase 1/2 studies demonstrated an acceptable safety profile and preliminary evidence of biological and clinical activity, although overall antitumour efficacy remained limited, highlighting the need for further optimisation and larger randomised clinical trials [54]. Collectively, these studies demonstrate that defined LBPs are feasible and safe, but additional clinical evidence is required before they can be incorporated into routine oncology practice. The major microbiome-targeted therapeutic strategies currently under investigation and their proposed mechanisms for enhancing anti-tumour immunity are summarised in Figure 2.

Despite these advances, several challenges remain. The mechanisms responsible for therapeutic efficacy are still incompletely understood, and microbial strains that demonstrate robust immunomodulatory effects in preclinical models often fail to reproduce comparable clinical benefits in humans. Microbial engraftment remains highly variable and may depend on baseline microbiome composition, diet, antibiotic exposure, host immunity, and ecological competition within the recipient microbiota [91]. This inter-individual variability may limit the reproducibility and durability of therapeutic responses. Furthermore, identifying the optimal microbial composition and, more importantly, the functional characteristics required to achieve consistent clinical benefit across different tumour types and therapeutic settings remains a major unresolved challenge.

## 5. Challenges and Future Directions in Microbiome-Guided Immunotherapy

### 5.1. Analytical Validation and Clinical Translation

Despite growing evidence linking the gut microbiome to ICI outcomes, its translation into clinical practice remains challenging. Microbiome-associated signatures frequently differ across studies because of variation in tumour type, patient population, geographical location, diet, medication exposure, treatment regimen, sampling procedures, and analytical methodology. Moreover, taxonomic associations alone neither establish causality nor adequately capture microbial function. Future studies should therefore prioritise harmonised sampling procedures, standardised analytical pipelines, longitudinal study designs, and prospective validation in geographically independent cohorts. A clinically useful microbiome biomarker will probably require the integration of microbial functional data with host, tumour and clinical characteristics. A prespecified composite microbiome activity score could combine strain-resolved metagenomic pathways, targeted microbial metabolites, intestinal barrier markers, circulating cytokines, immune-cell phenotypes, and established tumour variables. Feature selection should be performed exclusively within the training cohort, after which the model, thresholds, missing-data rules, and intended clinical application should be fixed before external validation. Longitudinal sampling should also determine whether changes in the score precede radiological response and whether intervention-induced microbial changes mediate clinical benefit. Separate predictive models may be required for treatment efficacy, toxicity and microbial engraftment because these outcomes may involve distinct biological mechanisms. Microbiome-directed interventions should similarly be evaluated through biomarker-guided clinical trials that link a measurable microbial or functional deficit to a biologically plausible intervention. Dietary modulation, FMT, and LBPs have demonstrated feasibility or early biological activity [18,50,51,52,53,54,55,56]; however, optimal patient and donor selection, durability of engraftment, manufacturing standards, regulatory requirements, and long-term safety remain unresolved [57,58,91,93]. Trials should include appropriate standard-of-care controls and distinguish microbial target engagement from clinical efficacy. Until reproducible biomarkers and clinical utility have been established, individual responder-associated taxa should not be interpreted as universal predictors or used to guide direct-to-consumer interventions. Progress towards microbiome-guided immunotherapy will therefore require analytical validation, mechanistic confirmation, prospective clinical evaluation and assessment of cost-effectiveness.

### 5.2. Contradictory Evidence and Context Dependence

A major challenge is that microbial features associated with favourable outcomes in one clinical setting may be absent or show the opposite association in another. Although *A. muciniphila*, *Faecalibacterium* spp. and members of the Ruminococcaceae are frequently enriched in ICI responders, these associations are not consistently reproduced across cohorts, and the same taxon may perform different functions depending on its strain, surrounding microbial community, diet, geography, and treatment context [9,10,11,15,16,65]. *R. bromii* illustrates this context dependence: it has been identified within favourable responder-associated communities in melanoma FMT studies, yet was enriched in non-responders in the PURE-01 urothelial cancer cohort [55,84]. Contradictory findings also extend beyond taxonomy. Higher microbial diversity is often associated with favourable outcomes, whereas indiscriminate probiotic supplementation may reduce diversity and has been associated with poorer responses despite the presumed beneficial nature of the administered microorganisms [18]. Similarly, SCFAs can support epithelial-barrier integrity and CD8^+^ T-cell memory, but higher systemic SCFA concentrations have also been associated with reduced benefit from CTLA-4 blockade [19,20]. These apparent contradictions do not invalidate microbiome-based biomarkers; rather, they demonstrate that taxonomic abundance alone is insufficient. Future studies should therefore evaluate strain-resolved function, metabolite concentration and anatomical compartment, tumour type, treatment regimen, and host covariates, with external validation required before any microbial signature is used clinically.

### 5.3. A Prospective Trial Design for Precision Microbiome Interventions

To move microbiome intervention research beyond small, unselected exploratory studies, we propose a prospective trial design based on predefined functional eligibility and sequential biological assessment. Patients would first undergo standard tumour specific assessment for ICI eligibility, followed by baseline metagenomic, metabolomic, dietary, medication and immune profiling. Entry into each microbiome intervention group would require a prespecified and potentially modifiable functional abnormality, rather than the presence or absence of an individual bacterial taxon. Participants with the same functional abnormality would then be randomised between the mechanism matched microbiome intervention and an appropriate control, while tumour type, ICI regimen and recent antibiotic exposure would be used as stratification variables [13,14].

Serial stool and blood samples should be collected before intervention, before ICI administration and at predefined early treatment time points. Tumour tissue should also be analysed when clinically feasible. Biological continuation criteria should be established in advance and should include engraftment where relevant, restoration of the targeted microbial pathway, alteration of the corresponding metabolite and induction of the predicted immune response. Clinical outcomes should then be evaluated independently using radiological response, progression free survival, overall survival, toxicity, and quality of life. Biological target engagement should support mechanistic interpretation but should not replace controlled assessment of clinical efficacy.

This design would permit different causes of treatment failure to be distinguished prospectively. Failure of engraftment could reflect inadequate microbial delivery, donor recipient incompatibility, or resistance from the existing microbial ecosystem. Engraftment without functional restoration would indicate that microbial presence alone was insufficient, while functional restoration without the predicted immune response would challenge the proposed mechanism. Immune modulation without clinical benefit could instead indicate persistent tumour intrinsic resistance. Applying the same core design principles to dietary modulation [18], LBPs and defined microbial consortia [50,51,52,53,54], and FMT [55,56] would facilitate comparison of their biological activity and provide a clearer route from patient selection to clinical validation. FMT trials should also incorporate rigorous donor screening and surveillance for pathogen transmission [57,58].

## 6. Conclusions

The rapid expansion of microbiome research has fundamentally advanced our understanding of host–tumour–immune interactions in cancer therapy. While precision oncology has traditionally focused on tumour genomics and targeted therapies, accumulating evidence demonstrates that the gut microbiome represents an additional and highly dynamic determinant of immunotherapy response. Through its influence on microbial metabolism, epithelial barrier integrity, antigen presentation, immune cell activation, and inflammatory signalling, the gut microbiome contributes to both the efficacy and toxicity of ICIs.

Although responder-associated taxa vary across tumour types and patient populations, current evidence increasingly points to microbial ecosystem structure and function as being more informative than individual microorganisms. In several cohorts, greater microbial diversity and enrichment of immunomodulatory and SCFA-producing bacteria have been associated with more favourable anti-tumour immunity and ICI outcomes. However, SCFAs do not exert uniformly beneficial effects; their activity depends on the specific metabolite, concentration, anatomical compartment, tumour metabolic state, and treatment context. Conversely, loss of microbial diversity, impaired epithelial barrier integrity, and persistent inflammatory signalling are more consistently associated with immune dysfunction and resistance to immunotherapy.

Importantly, the gut microbiome represents a potentially modifiable factor capable of influencing systemic immunity and the tumour microenvironment. This has stimulated the development of microbiome-targeted therapeutic strategies, including dietary modulation, FMT, and LBPs. Among these approaches, responder-derived FMT currently provides the strongest clinical proof-of-concept that microbiome modulation can restore responsiveness to ICIs in selected patients, while next-generation microbial therapeutics aim to reproduce these benefits through standardised, mechanism-based interventions.

Collectively, these findings support a conceptual shift in cancer immunotherapy from viewing the microbiome as a descriptive biomarker to recognising it as a therapeutically actionable component of the host immune ecosystem. At the same time, the field is evolving beyond the identification of individual bacterial taxa towards understanding microbial functions, metabolic pathways, ecological interactions, and host–microbiome communication as the principal drivers of therapeutic response.

Despite these advances, clinical implementation will require continued validation of predictive biomarkers, standardised analytical methodologies, and robust evidence from large perspective randomised clinical trials. Integrating microbiome science with immunology, microbial ecology, metabolomics, and other multi-omics approaches will further improve our understanding of the complex interactions governing host responses to immunotherapy.

In conclusion, the gut microbiome has emerged as a critical regulator of cancer immunotherapy outcomes and represents a promising target for therapeutic intervention. As mechanistic understanding continues to advance and clinical evidence matures, microbiome-guided strategies have the potential to become an integral component of precision oncology, enabling more personalised treatment approaches, overcoming resistance to immune checkpoint inhibitors, and ultimately improving long-term patient outcomes.

## Figures and Tables

**Figure 1:**
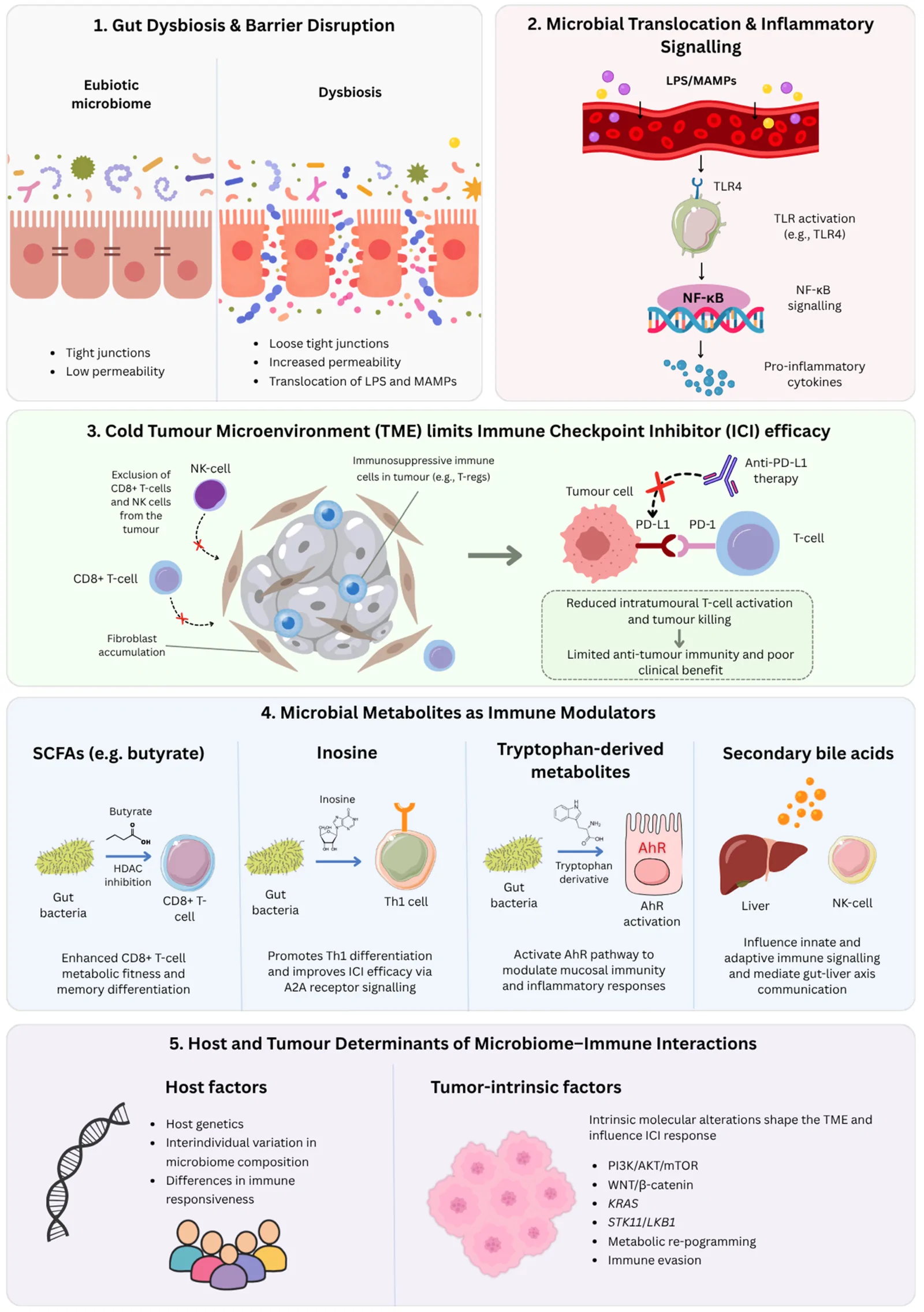
Mechanistic overview of gut microbiome-mediated regulation of cancer immunotherapy. Dysbiosis can disrupt barrier integrity and promote microbial-product translocation, TLR4/NF-κB signalling, chronic inflammation, and an immune-excluded tumour microenvironment. Microbial metabolites—including SCFAs, inosine, tryptophan derivatives, and secondary bile acids—can modulate T-cell differentiation, antigen presentation, and inflammatory signalling. These effects are conditional on host and tumour biology: restoration of microbial function may improve immune competence, but tumour-intrinsic defects such as *JAK1/2* loss, impaired antigen presentation, or immune-exclusion pathways can still prevent ICI response. The red cross indicates therapeutic blockade of the PD-1/PD-L1 interaction. Created using Canva Pro (Canva Pty Ltd., Melbourne, Australia).

**Figure 2:**
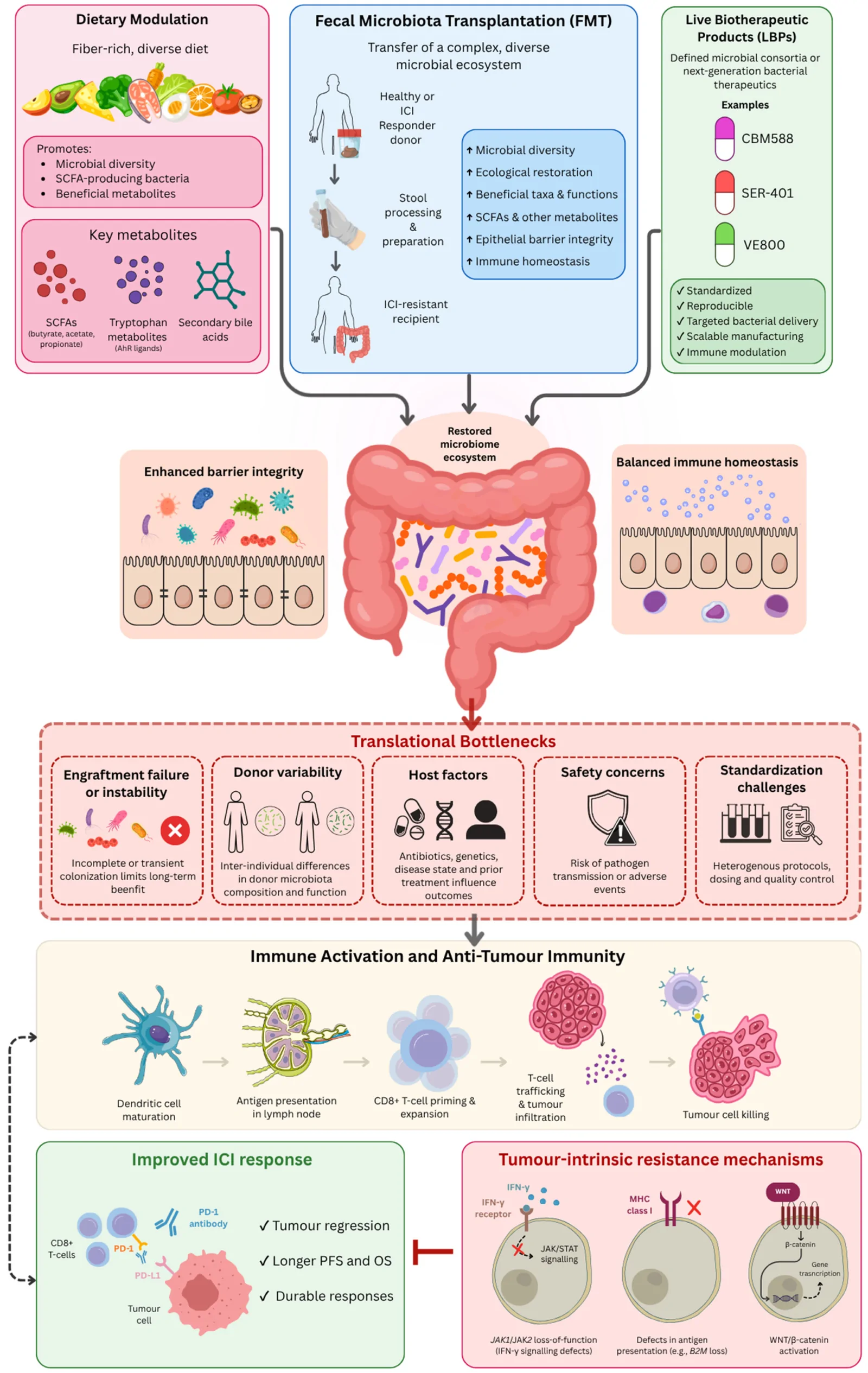
Microbiome-targeted strategies and their potential role in improving ICI response. Dietary modulation, FMT, and LBPs aim to restore a diverse and functionally beneficial gut microbiome, enhancing microbial metabolites, epithelial barrier integrity, and immune homeostasis. However, clinical translation is limited by engraftment instability, donor-host variability, host factors, safety concerns, and challenges in standardization. Successful microbiome restoration may promote dendritic cell maturation, antigen presentation, CD8^+^ T-cell priming, and tumour infiltration, thereby enhancing anti-tumour immunity and ICI response. Conversely, tumour-intrinsic resistance mechanisms, including impaired IFN-γ/JAK–STAT signalling, defective antigen presentation, and WNT/β-catenin activation, may limit therapeutic efficacy. Arrows indicate the direction of the proposed biological or therapeutic effects; crosses indicate failure, disruption, or loss of function, and the blunt-ended line indicates inhibition of ICI response by tumour-intrinsic resistance mechanisms. Created by the authors using Canva Pro (Canva Pty Ltd., Melbourne, Australia).

**Table 1:** Comparative summary of the strength, key findings, and limitations of current microbiome–ICI evidence across tumour types.

Tumour Type	Evidence	Main Synthesis	Key Limitation	Key References
Melanoma	Highest	Cross-cohort associations and early responder-derived FMT studies support a relationship between the gut microbiome and ICI response.	Microbial signatures show limited reproducibility across cohorts, and FMT studies remain relatively small.	[15,55,56]
RCC	Moderate; emerging interventional evidence	Observational evidence has been complemented by encouraging findings from CBM588 supplementation and responder-derived FMT.	Trials remain small, and combination regimens make the independent microbiome effect difficult to determine.	[50,77,79]
NSCLC	Moderate; predominantly observational	Antibiotic exposure and *A. muciniphila* have been associated with ICI outcomes, with emerging evidence from FMT.	Most evidence remains associative, and clinical and treatment-related confounding is substantial.	[13,65,73]
HCC	Early	The gut–liver axis and microbiota-dependent bile-acid metabolism provide mechanistic plausibility, supported by initial clinical associations.	Cirrhosis, disease aetiology and hepatic dysfunction strongly influence microbial and immune variation; interventional validation remains limited.	[23,74,75]
CRC	Context-dependent	Evidence is stronger for microbial contributions to carcinogenesis and tumour immune regulation than for prediction of ICI response.	dMMR/MSI-H status remains a substantially better-established predictor of ICI benefit than any microbiome signature.	[5,62,63]
Urothelial/bladder cancer	Exploratory	Urinary, tissue and stool microbiome associations have been reported, including a stool signature associated with neoadjuvant pembrolizumab response.	Low biomass, sampling heterogeneity and differences among microbial compartments limit reproducibility.	[81,82,84]

**Table 2:** Clinical and observational evidence linking microbiome profiles and microbiome-directed interventions to ICI outcomes across solid tumours. Findings should be interpreted within each tumour and treatment context and do not represent universal microbial biomarkers. FMT, faecal microbiota transplantation; ICI, immune checkpoint inhibitor; LBP, live biotherapeutic product; NSCLC, non-small-cell lung cancer; RCC, renal cell carcinoma; HCC, hepatocellular carcinoma; CRC, colorectal cancer; UC, urothelial carcinoma.

Cancer	Trial/Study	Modality	Responder-Associated Microbial Signatures	Non-Responder-Associated Microbial Signatures	References
Melanoma	NCT03341143	FMT from PD-1 responders	Species: *F. prausnitzii*, *Eubacterium rectale*, *Anaerostipes hadrus*, *R. bromii*, *B. longum*, *B. adolescentis* Phyla: Firmicutes, Actinobacteria	Species: *E. coli*, *Klebsiella pneumoniae* Phyla: Proteobacteria	[55]
	NCT03353402	Responder-derived FMT + PD-1 re-induction	Species: Enrichment of donor-like SCFA-producing Firmicutes and *Bifidobacterium*; increased diversity	Persistence of dysbiotic, Proteobacteria-rich communities	[56]
	NCT03772899_MIMic	FMT from rational donors	Species: *E. rectale*, *E. ramulus*, *F. prausnitzii*, *R. obeum*, *B. longum* Phyla: Firmicutes, Actinobacteria	Species: *Hungatella hathewayi* Phyla: Proteobacteria	[59]
	NCT03817125_SER401	Defined Firmicutes spore consortium	Baseline: High Ruminococcaceae (Firmicutes) associated with better immune tone	Baseline: Antibiotic-disrupted, low-diversity communities	[52]
	NCT03775850_EDP1503	Live biotherapeutic (*B. animalis* subsp. *lactis*) + pembrolizumab	None reported (no microbiome profiling)	Not reported	[54]
	Observational cohort	Baseline microbiome → ICI response	Features: High diversity; enriched *Faecalibacterium* and Firmicutes	Features: Low diversity; enriched Proteobacteria and oral pathobionts	[10]
	Observational cohort	Baseline microbiome → ICI response	Species: *B. thetaiotaomicron*, *F. prausnitzii*, *Collinsella aerofaciens*	Species: *B. fragilis*; depleted SCFA producers	[11]
NSCLC, melanoma	NCT04951583_LUMINate	Healthy-donor FMT + anti-PD-1 (NSCLC) or anti-PD-1/anti-CTLA-4 (melanoma)	Distinct post-FMT microbiome composition; greater loss of baseline bacterial species	Frequent persistence of baseline *Enterocloster citroniae*, *E. lavalensis* and *C. innocuum*	[73]
NSCLC, RCC	Translational microbiome study	FMT from ICI responders	Species: *A. muciniphila*, *Barnesiella intestinihominis*, *Enterococcus faecium* Phyla: Verrucomicrobia, Bacteroidetes, Firmicutes	Species: Enterobacteriaceae (*Escherichia*, *Klebsiella*) Phyla: Proteobacteria	[9]
Solid tumours (NSCLC, RCC, bladder cancer and melanoma)	NCT03637803	MRx0518 live biotherapeutic + pembrolizumab	Results not reported in the cited trial record	Results not reported in the cited trial record	ClinicalTrials.gov NCT03637803
	NCT05008861	FMT + PD-1/PD-L1 rechallenge	Results pending	Results pending	ClinicalTrials.gov NCT05008861
Colorectal cancer	NCT05250635	FMT + PD-1 inhibitor + VEGFR TKI	Results pending	Results pending	ClinicalTrials.gov NCT05250635
	NCT02060188_CheckMate-142	ICI monotherapy (no directed microbiome modulation)	No specific gut taxa formally reported as responders	No specific taxa reported	[60]
	NCT01876511	ICI monotherapy	Not reported	Not reported	[61]
Hepatocellular carcinoma	NCT05032014	Probio-M9 + PD-1 therapy	Results not reported	Results not reported	ClinicalTrials.gov NCT05032014
Renal Cell carcinoma	NCT04163289 (PERFORM)	Healthy-donor encapsulated FMT + ICI-based therapy	Improved alpha diversity and durable engraftment of anti-inflammatory taxa/functions correlated with response and reduced toxicity	*Segatella copri* expansion was associated with grade ≥ 3 immune-related adverse events	[78]
	NCT04758507 (TACITO)	Selected responder-donor FMT + pembrolizumab/axitinib	Donor-strain engraftment, increased alpha diversity and larger community shifts; acquisition or loss of specific strains was associated with outcome	No validated non-responder species panel reported	[79]
	NCT05273255	FMT + ICI	Results pending	Results pending	ClinicalTrials.gov NCT05273255
Urothelial carcinoma	NCT02736266 (PURE-01 microbiome analysis)	Neoadjuvant pembrolizumab; exploratory stool profiling	*Sutterella* enriched in pathological responders	*R. bromii* enriched in non-responders	[84]
Melanoma	NCT04988841 (PICASSO)	MaaT013 pooled-donor microbiota + ipilimumab/nivolumab	Results not reported in the cited trial record	Results not reported in the cited trial record	ClinicalTrials.gov NCT04988841
	NCT05286294 (MITRIC)	Responder-derived FMT + continued ICI	Engraftment occurred in most recipients; one patient experienced clinical benefit lasting more than six months.	No objective responses were observed; overall clinical activity was limited, and no validated taxonomic response signature was identified.	[92]

## Data Availability

No new data were created or analyzed in this study. Data sharing is not applicable to this article.

## Abbreviations

The following abbreviations are used in this manuscript:

ACSS2	Acyl-CoA synthetase short-chain family member 2
AhR	Aryl hydrocarbon receptor
APCs	Antigen-presenting cells
ARG1	Arginase 1
CART-T	Chimeric antigen receptor T Cell
CBM588	*Clostridium butyricum* MIYAIRI 588
CCL20	C-C motif chemokine ligand 20
CRC	Colorectal cancer
CTLA-4	Cytotoxic T-lymphocyte-associated protein 4
DCs	Dendritic cells
FFAR2	Free fatty acid receptor 2
FFAR3	Free fatty acid receptor 3
FMT	Faecal microbiota transplantation
Gαq	G protein subunit alpha q
HCC	Hepatocellular carcinoma
HDAC	Histone deacetylase
ICIs	Immune checkpoint inhibitors
ID2	Inhibitor of DNA binding 2
IFN-γ	Interferon gamma
IGF1	Insulin-like growth factor 1
IL	Interleukin
*JAK1/2*	Janus kinase 1/2
LBPs	Live biotherapeutic products
LPS	Lipopolysaccharide
MAMPs	Microbe-associated molecular patterns
MAPK	Mitogen-activated protein kinase
MART-1	Melanoma antigen recognised by T cells 1
MDSCs	Myeloid-derived suppressor cells
MHC	Major histocompatibility complex
MSI-H	Microsatellite instability-high
mTOR	Mechanistic target of rapamycin
NF-κB	Nuclear factor kappa B
NK	Natural Killer
NSCLC	Non-small cell lung cancer
OS	Overall survival
PD-1	Programmed cell death protein 1
PD-L1	Programmed death-ligand 1
PFS	Progression-free survival
PI3K	Phosphoinositide 3-kinase
PPARγ	Peroxisome proliferator-activated receptor gamma
PRRs	Pattern recognition receptors
RCC	Renal cell carcinoma
SCFAs	Short-chain fatty acids
TKI	Tyrosine kinase inhibitor
TLR3	Toll-like receptor 3
TLR4	Toll-like receptor 4
TLR5	Toll-like receptor 5
TME	Tumour microenvironment
TNF-α	Tumour necrosis factor alpha
TRAMP	Transgenic adenocarcinoma of the mouse prostate
Tregs	Regulatory T cells
